# *Aggregatibacter actinomycetemcomitans* Cytolethal Distending Toxin Induces Cellugyrin-(Synaptogyrin 2) Dependent Cellular Senescence in Oral Keratinocytes

**DOI:** 10.3390/pathogens13020155

**Published:** 2024-02-08

**Authors:** Bruce J. Shenker, Jonathan Korostoff, Lisa P. Walker, Ali Zekavat, Anuradha Dhingra, Taewan J. Kim, Kathleen Boesze-Battaglia

**Affiliations:** 1 Department of Basic and Translational Sciences, School of Dental Medicine, University of Pennsylvania, Philadelphia, PA 19104, USA; lism@dental.upenn.edu (L.P.W.); seyed20@dental.upenn.edu (A.Z.); dhingra@upenn.edu (A.D.); battagli@dental.upenn.edu (K.B.-B.); 2 Department of Periodontics, School of Dental Medicine, University of Pennsylvania, Philadelphia, PA 19104, USA; jkorosto@upenn.edu (J.K.); taewank@upenn.edu (T.J.K.)

**Keywords:** *Aggregatibacter actinomycetemcomitans*, cytolethal distending toxin, senescence, oral keratinocytes, cellugyrin, synaptogyrin-2, periodontal disease

## Abstract

Recently, we reported that oral-epithelial cells (OE) are unique in their response to *Aggregatibacter actinomycetemcomitans* cytolethal distending toxin (Cdt) in that cell cycle arrest (G2/M) occurs without leading to apoptosis. We now demonstrate that Cdt-induced cell cycle arrest in OE has a duration of at least 7 days with no change in viability. Moreover, toxin-treated OE develops a new phenotype consistent with cellular senescence; this includes increased senescence-associated β-galactosidase (SA-β-gal) activity and accumulation of the lipopigment, lipofuscin. Moreover, the cells exhibit a secretory profile associated with cellular senescence known as the senescence-associated secretory phenotype (SASP), which includes IL-6, IL-8 and RANKL. Another unique feature of Cdt-induced OE senescence is disruption of barrier function, as shown by loss of transepithelial electrical resistance and confocal microscopic assessment of primary gingival keratinocyte structure. Finally, we demonstrate that Cdt-induced senescence is dependent upon the host cell protein cellugyrin, a homologue of the synaptic vesicle protein synaptogyrin. Collectively, these observations point to a novel pathogenic outcome in oral epithelium that we propose contributes to both *A. actinomycetemcomitans* infection and periodontal disease progression.

## 1. Introduction

*Aggregatibacter actinomycetemcomitans* has been linked to systemic disorders, including endocarditis and brain abscesses, among others [1]. In the oral cavity, the organism has long been associated with what prior to 2017 was referred to as localized aggressive periodontitis (LAP), a complex disorder involving risk factors of both host and microbial origin [2,3,4,5,6,7,8]. Although LAP no longer exists as a disease classification, the clinical manifestations remain part of the new classification corresponding to aggressive molar-incisor pattern periodontitis (MIPP). *A. actinomycetemcomitans* was previously thought to function as the causative agent of LAP. It is now believed that the initial colonization of supragingival biofilms by *A. actinomycetemcomitans* represents a risk factor for the onset of gingival inflammation. As an accessory pathogen, *A. actinomycetemcomitans* eventually translocates from the gingival margin through the gingival epithelium into the underlying connective tissue, a process associated with the conversion from health to disease [1,2,6]. In this regard, *A. actinomycetemcomitans* exhibits virulence properties that contribute to this new role as an accessory pathogen; these include tissue invasiveness, the ability to create an environment facilitating the accumulation of other organisms, evasion or subversion of host defenses and the capacity to promote inflammation. Collectively, these pathogenic attributes contribute to the accumulation of inflammophilic organisms that mediate downstream events in the pathogenesis of periodontitis (reviewed in [1,2,7]. Importantly, *A. actinomycetemcomitans* expresses two exotoxins, cytolethal distending toxin (Cdt) and leukotoxin [1,9,10,11], which are capable of subverting host cell function [1,2,8,12].

*A. actinomycetemcomitans* cyolethal distending toxin (*Aa*Cdt) is encoded by three genes, cdtA, cdtB, and cdtC, encoding three polypeptides designated as CdtA, CdtB and CdtC with molecular masses of 23–30, 28–32 and 19–20 kDa, respectively. The three proteins associate with one another to form a holotoxin [10,13,14,15,16,17] that functions as an AB2 toxin. Subunits CdtA and CdtC comprise the cell-binding (B) component, and CdtB is the active subunit. It is noteworthy that Cdts represent a highly distributed and conserved family of putative virulence factors produced by more than 30 γ- and ε-Proteobacteria, which are responsible for chronic infections and inflammatory diseases that typically affect mucocutaneous tissue (reviewed in [11]). Cdts, regardless of bacterial origin, cause similar effects on proliferating cells: cell cycle arrest (typically G2/M) and eventually apoptotic cell death [10,14,18,19,20,21,22,23,24,25,26]. Recent observations suggest that Cdt is also capable of inducing functional alterations in the absence of cell death in non-proliferating cells [27,28,29,30].

We propose that Cdt contributes to several virulence properties of *A. actinomycetemcomitans* as it functions as a tri-perditious toxin that affects lymphocyte, macrophage, mast cell and epithelial function, thereby altering acquired and innate immunity as well as epithelial barrier integrity [11,31]. Cdt is able to exhibit these diverse effects and intoxicate multiple cell types by virtue of three unique properties that we have identified: (1) exploitation of a ubiquitous cell receptor, cholesterol [32,33,34]; (2) utilization of a novel host protein, cellugyrin, for cell entry and trafficking [35,36], and (3) utilization of a molecular mode of action that disrupts phosphatidylinositol-3 kinase (PI-3K) signaling, a pathway utilized by virtually all cells to regulate an array of functions [27,29,37,38].

Recently, we demonstrated that oral-epithelial cells (OE) are unique in their response to *Aa*Cdt [39]. Assessment of the effect of *Aa*Cdt on two immortalized OE lines as well as primary gingival keratinocytes (PGKs) showed that all three cell types were sensitive to *Aa*Cdt-induced cell cycle arrest as toxin-treated cells accumulated in the G2/M phase within 24 h of exposure to low Cdt concentrations (pg/mL) [39]. Similar to other cell types, toxin-treated cells also exhibited PI-3K signaling blockade, leading to glycogen synthase kinase 3β (GSK3β) activation, a requirement for Cdt-induced cell cycle arrest. However, unlike other cells, which typically undergo apoptosis following Cdt-induced cell cycle arrest, epithelial cell G2/M arrest did not lead to cell death [39]. We have now extended these observations and demonstrated that Cdt-induced cell cycle arrest in OE is durable. Moreover, OEs develop a phenotype typical of senescent cells, increased senescence-associated β-galactosidase (SA-β-gal) activity, accumulation of the lipopigment, lipofuscin, a product of oxidative processes, and the senescence-associated secretory phenotype (SASP), typified by enhanced production of IL-6, IL-8, and RANKL. We also show that senescent OEs exhibit a breakdown in barrier function, a unique characteristic of senescent cells. Finally, we determined that Cdt-induced cellular senescence is dependent upon the host cell protein cellugyrin. Collectively, these observations point to a novel pathogenic outcome in the oral epithelium that likely contributes to altered epithelial integrity and thereby promotes both *A. actinomycetemcomitans* infection and disease progression.

## 2. Materials and Methods

### 2.1. Oral Keratinocyte Culture and Gene Editing

The TIGK cell line was established from human gingival epithelial cells and immortalized with bmi1-transduction followed by human telomerase reverse transcriptase (hTERT) (kindly provided by RJ Lamont) [40]. TIGK cells were incubated in DermaLife K Basal Medium (Lifeline Cell Technology, Frederick, MD, USA) supplemented with glutamine, extract P, epinephrine, rh TGF, hydrocortisone hemisuccinate, rh insulin, apo-transferrin and calcium chloride.

Primary human gingival keratinocytes (PGK) were derived from discarded healthy gingival tissue obtained from patients undergoing crown lengthening procedures as previously described [39,41]. Briefly, tissue was washed in a HAMS F12 nutrient mixture (ThermoFisher Scientific, Waltham, MA, USA) containing 1% penicillin/streptomycin and 1% ampthotericin and then cut into 0.3 cm^2^ fragments. The pieces of gingiva were incubated in HAMS F12 medium described above and also containing 2% dispase (Sigma Aldrich Co.; Burlington, MA, USA) for 24 h at 37 °C. Tissue was then separated with vigorous pipetting in 0.05% trypsin/EDTA (Life Technologies; Carlsbad, CA, USA), and the cell suspension was centrifuged, resuspended and incubated in Keratinocyte serum free medium (K-SFM; Life Technologies) containing bovine pituitary extract (BPE) (5 ng/mL), epidermal growth factor (10 μg/mL), and 2% penicillin/streptomycin.

We employed pLentiCRISPR V2 to generate cellugyrin-deficient TIGK cells (TIGK^Cg−^) [39]. Briefly, two separate CRISPR-Cas9 guide sequences for cellugyrin were inserted into plentiCRISPR V2 plasmids, in which the puromycin resistance cassette was replaced with a neomycin resistance cassette (GTACATCTGCTTAGACTCGT, CGCGTCGACCACCAAGAAGA) (Genscript, Piscataway, NJ, USA). Plasmids were co-transfected into HEK-293T cells, and viral supernatants were collected for introduction into cells. Virus was added to cells at a MOI of 4 and incubated overnight; cells were washed, then incubated for an additional 24 h before being selected with 250 ng/mL neomycin. Cells were plated for single-cell cloning, and cellugyrin-negative lines were generated from selected clones.

### 2.2. Preparation of Cdt

The construction and expression of the plasmid containing the *cdt* genes for the holotoxin (pUCAacdtABChis) have previously been reported [42]. The histidine-tagged holotoxin was isolated by nickel affinity chromatography, as previously described [15].

### 2.3. Assessment of Cell Proliferation

To measure Cdt-induced cell cycle distribution, replicate cultures of TIGK cells (10^5^) were incubated for the time indicated, harvested (TIGK cells required trypsin digestion), washed, and fixed for 60 min with cold 80% ethanol [43]. Cells were stained with 10 µg/mL propidium iodide containing 1 mg/mL RNase (Sigma Aldrich Co.) for 30 min. Samples were analyzed on a Becton-Dickinson LSRII flow cytometer (BD Biosciences; San Jose, CA, USA); a minimum of 15,000 events were collected for each sample. Cell cycle analysis was performed using Modfit 6.0 (Verity Software House; Topsham, ME, USA).

Bromodeoxyuridine (BrdU) incorporation was assessed in TIGK cultures set up as described above with and without Cdt for 72 h. BrdU (10 μM; BrdU Kit; Thermofisher) was added to TIGK cultures for the last 4 h of incubation. Cells were harvested, fixed in 80% EtOH for 30 min and then treated with 2N HCl containing 0.5%Triton X-100. Following centrifugation, cells were resuspended in 0.1 M Na_2_B_4_O_7_ for 5 min, and then in PBS containing 2% BSA and 0.05% Tween 20 (BioRad; Hercules, CA, USA). Cells were stained with anti-BrdU-FITC (Invitrogen, Waltham, MA, USA) for 1 h at RT, washed and stained with propidium iodide. Cells were analyzed by multiparametric flow cytometry on a Becton-Dickinson LSRII flow cytometer (BD Biosciences; San Jose, CA, USA); fluorochromes were excited with a 488 nm laser, and fluorescence was detected using a 530/30 filter (FITC) and a 575/26 filter (propidium iodide). A minimum of 15,000 events were collected for each sample, and analysis was performed using WinList 9.0.1 (Verity Software House).

Analysis of cell proliferation was also assessed on TIGK cells using ViaFluor 488 (Biotium; Fremont, CA, USA). Cell cultures were established in 24-well plates, as described above; cells were pre-treated with medium alone or medium containing Cdt (20 pg/mL) for 16 h. ViaFluor was added to each well for 15 min, according to the manufacturer’s directions after which cells were washed and incubated for 72 h. Cells were then harvested by trypsinization and analyzed for fluorescence as described above.

### 2.4. Assessment of SA-β-Gal Activity

TIGK cells (10^5^) were added to 24 well plates in the media described above; cells received media or Cdt for the time indicated. SA-β-gal activity was measured using the Senescence β-galactosidase activity assay kit (Cell Signaling, Danvers, MA, USA) as described by the manufacturer. Briefly, media was removed from cells and bafilomycin (to prevent acidification) was added for 1 h; the cell permeable fluorogenic substrate was added and the cells incubated for an additional 4 h at 37 °C. Cells were trypsinized and washed. Fluorescence resulting from hydrolysis of the substrate was analyzed by flow cytometry using a 488 nm laser and fluorescence emission assessed through a 530/30 nm filter. At least 10,000 events were analyzed.

### 2.5. Assessment of Lipofuscin Content

TIGK cells were established in 24-well plates as described above; at the times indicated, cells were stained using the SenTraGor reagent according to the manufacturer’s recommendation (Lab Supplies Scientific; Athens, Greece). Briefly, media was removed and cells harvested following trypsinization. Cells were fixed in 4% formaldehyde at RT for 20 min, washed, permeabilized in 0.01% Triton X-100 for 15 min, washed sequentially in 50% and 70% EtOH, and then incubated for 8 min with SenTraGor. Cells were washed with 50% EtOH and then stained with anti-biotin antibody conjugated to AF488 (Santa Cruz Biotechnology; Santa Cruz, CA, USA) or isotype (IgG1) control antibody (Santa Cruz Biotechnology) for 1 h at RT; after washing, cells were analyzed by flow cytometry using a 488 nm laser. Fluorescence emission was measured through a 530/30 filter; at least 10,000 cells were analyzed.

### 2.6. Analysis of Cytokine Release from TIGK Cells

TIGK cultures were set up as described above; cytokine production was measured in TIGK supernatants 72 h after exposure to medium with or without Cdt. In some experiments, cells were pre-exposed to necrosulfonamide [(NSA); Millipore Sigma, Burlington, MA, USA] for one h prior to the addition of Cdt. Culture supernatants were collected and analyzed by ELISA for IL-6, IL-8 and RANKL using commercially available kits according to the manufacturer’s instructions (Peprotech; Cranberry, NJ, USA) [27]. In each instance, the amount of cytokine present in the supernatant was determined using a standard curve.

### 2.7. Analysis of Epithelial Barrier Integrity

Assessment of Trans-Epithelial Electrical Resistance (TEER) is a widely accepted quantitative technique to measure the integrity of the barrier function of OE culture models [44]. Polarized PGKs (1.5 × 10^5^) were grown on 12-well transwell inserts (Corning; Corning, NY, USA) in the medium described above. Cells were grown to confluence and monitored for the establishment of TEER with the STX2 electrode (Epithelial Volt/Ohm Meter; World Precision Instruments, Sarasota, FL, USA). Cdt or media were then added to cultures, and TEER was measured at 24 h intervals.

PGKs were grown as above on transwell dishes and treated with Cdt (10 pg/mL, 48 h) or medium only, washed in PBS, and fixed in 4% PFA for 15 min at room temperature. The cells were permeabilized and blocked in 5% BSA and 0.2% Triton X-100 in PBS (PBST) at 37 °C for 1 h. Cells were then incubated with a 1:100 dilution of anti-ß-catenin antibody (Cell Signaling) diluted in blocking solution at 4 °C overnight, washed three times with PBST, incubated with donkey anti-rabbit Alexa Fluor 488 (1:1000; Invitrogen) and Hoechst 33258 (1:10,000; Invitrogen) at 37 °C for 1 h, washed and mounted in Prolong Gold Antifade reagent (P36930, Invitrogen). Confocal Z-stack images were acquired on a Nikon A1R laser scanning confocal microscope with a 60X (water) objective at 18 °C, and the data were analyzed using Nikon software (NIS Elements AR 5.30.03) [30].

## 3. Results

### 3.1. Cdt Induces a Senescent Cell Phenotype in Oral Epithelial Cells

**Figure 1:**
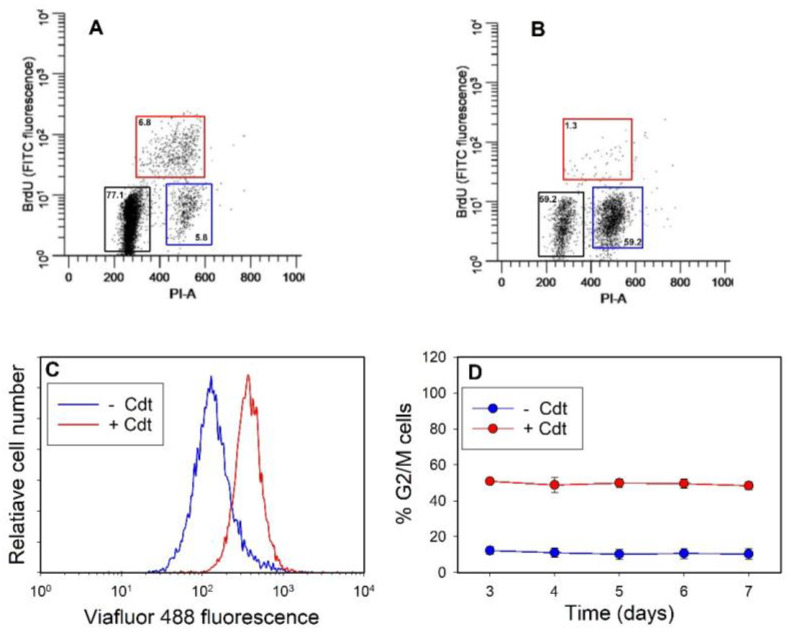
Cdt induces durable cell cycle arrest in OE. TIGK cells were treated with Cdt for varying periods of time as indicated and then monitored for cell cycle arrest. Panels (**A**) (control cells) and (**B**) (Cdt-treated cells) show the effect of Cdt (20 pg/mL) on TIGK proliferation after 72 h. Cell cycle progression was assessed using dual parameter flow cytometry; DNA content was assessed by monitoring propidium iodide fluorescence (PI-A) and incorporation of BrdU-FITC. Boxes indicate gates for cell cycle analysis: G1/G0 (black), G2/M (blue) and S (red); numbers indicate the percentage of cells within each gated box. Panel (**C**) shows the analysis of cell cycle progression in control (blue line) and toxin-treated (red line) cells stained with Viafluor 488 and incubated for 72 h. The results for panels (**A**–**C**) are each representative of three experiments. Panel (**D**) shows cell cycle analysis of TIGK cells treated with medium (blue) and Cdt [20 pg/mL; (red)] for 3–7 days using propidium iodide. Cells were analyzed for cell phase as described in Section 2; the percentage of G2/M cells is plotted versus time. Results are compiled from three experiments and represent the mean ± SEM; all data points for Cdt-treated cells are significantly different from those observed in control cells (*p* < 0.01). Individual cell cycle histograms are shown in Figure S1.

We previously reported that the oral keratinocyte cell lines TIGK and OKF6 as well as PGKs exposed to Cdt exhibited cell cycle arrest (G2/M) that persisted for at least 72 h; notably, the arrest occurred in the absence of any evidence of DNA damage or apoptotic cell death [39]. In those studies, cell cycle arrest was determined by assessing DNA content with propidium iodide to measure cell distribution within the cell cycle. In our current study, we have expanded the assessment of Cdt-induced growth arrest by first utilizing dual parameter flow cytometric analysis to simultaneously measure G0/G1 and G2/M with propidium iodide and more accurately assess the S-phase with bromodeoxyuridine (BrdU), a synthetic nucleoside analogue with a chemical structure similar to thymidine. Representative results are shown in Figure 1; cells were exposed to Cdt or medium alone for 72 h. BrdU was added to the cultures for the last 4 h; 6.8% of untreated (no toxin) TIGK cells exhibited positive BrdU fluorescence (Figure 1A). During the same time period, cells treated with 20 pg/mL Cdt exhibited fewer S-phase cells, as only 1.3% were BrdU positive (Figure 1B); these results further support the conclusion that exposure of OEs to Cdt leads to growth arrest. Consistent with earlier studies, Cdt treatment resulted in an increase in the percentage of G2/M cells to 59.2% relative to untreated cells, which contained 5.8% G2/M cells. The percentage of cells in G0/G1 decreased from 77.1% in control cells to 32.6% in cells treated with Cdt.

In a complementary approach, we utilized Viafluor 488 to assess the effect of Cdt on cell proliferation. Cells were treated with Cdt (or medium) and then labeled with Viafluor as described in Section 2. Fluorescence was assessed 72 h later; each round of cell division results in a reduction in fluorescence as the probe is distributed to daughter cells. Control and toxin-treated cells were compared based upon their level of fluorescence and designated as either high Viafluor fluorescence (Viafluor^high^) or low Viafluor fluorescence (Viafluor^low^). Control TIGK cells progressed through the cell cycle, resulting in a transition to a population characterized as Viafluor^low^ [mean channel fluorescence (MCF) 135] when compared to Cdt-treated TIGK cells (Figure 1C). The toxin-treated cells underwent cell cycle arrest, which was reflected in their retaining higher levels of Viafluor and hence exhibited brighter fluorescence given that the dye was not diluted out into daughter cells; the Viafluor^high^ exhibited a MCF of 430.

In a third assessment, the durability of Cdt-induced cell cycle arrest was analyzed using propidium iodide to determine cell cycle distribution at days 3–7-post toxin treatment (Figure 1D). We previously reported that Cdt-treated TIGK cells exhibited G2/M arrest as early as 24 h, and the percentage of G2/M cells remained elevated at 72 h. In our current analysis, the proliferating TIGK control cells routinely contained 10.3 ± 2.8 to 12.2 ± 2.1% G2/M cells; this is consistent with a distribution of normal proliferating cells (see Figure S1 for cell cycle profiles). In contrast, the percentage of G2/M Cdt-treated cells increased to 50.9 ± 2.1% at day 3, as previously reported [39]; the accumulation of G2/M cells remained at this level throughout days 4–7 of analysis. Collectively, these experiments indicate that OEs exposed to Cdt undergo a sustained, or durable, cell cycle arrest in the G2/M phase of the cell cycle.

**Figure 2:**
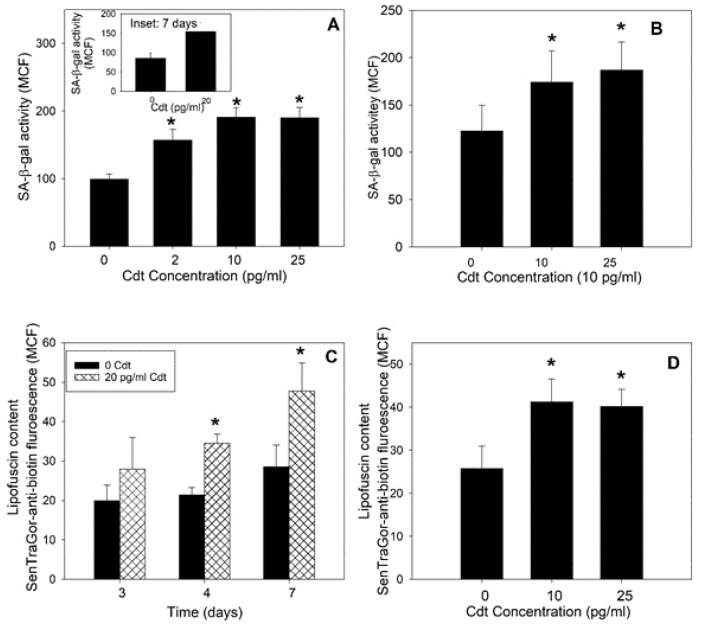
Cdt induces a cellular senescent phenotype in OEs characterized by increases in both SA-β-gal activity and lipofuscin content. TIGK cells were treated with 0–25 pg/mL Cdt for 72 h and then analyzed for SA-β-gal activity as described in Section 2. Data are plotted as fluorescence (MCF) vs. Cdt concentration. The inset (panel (**A**)) shows results for exposure to Cdt for 7 days. Panel (**B**) shows the effect of Cdt on SA-β-gal activity in toxin-treated PGK cells following 3 days of exposure to Cdt. Panel (**C**) shows the effect of Cdt (20 pg/mL) on lipofuscin levels in TIGK cells following 3–7 days of exposure to toxin. Lipofuscin was detected using biotinylated SenTraGor, followed by staining with an anti-biotin antibody conjugated to AF488. Results are plotted as fluorescence (MCF) versus time of exposure to Cdt (days). Panel (**D**) shows the effect of Cdt on lipofuscin levels in PGK cells after 4 days of treatment with toxin. Results are plotted as the MCF (mean ± SEM) of three experiments; * indicates statistical significance (*p* < 0.05).

Durable cell cycle arrest in an otherwise replication-competent cell is a critical feature of cellular senescence but not necessarily a defining feature alone. Cellular senescence is also associated with the acquisition of a new phenotype characterized by other common traits such as increased SA-β-gal activity and lipofuscin accumulation [45,46,47]. Therefore, we next utilized flow cytometry to assess Cdt-treated cells for changes in SA-β-gal activity by monitoring the hydrolysis of a cell-permeable fluorogenic substrate (see Section 2). As shown in Figure 2A, cells treated with Cdt for 72 h exhibited a significant dose-dependent increase in SA-β-gal activity, as the MCF increased to 156.7 ± 16.5 (2 pg/mL Cdt) and 191.1 ± 13.8 (10 pg/mL Cdt); this compares to a MCF of 99.5 ± 7.5 in control cells. Exposure to 25 pg/mL Cdt did not result in any further increase in SA-β-gal activity as the MCF was 190.4 ± 14.8. It should be noted that these levels remained elevated for 7 days (see Figure 2A inset). Experiments were also conducted with PGKs, which exhibited similar elevations in SA-β-gal activity (Figure 2B) when treated with 10 pg/mL (MCF 174 ± 33) and 25 pg/mL Cdt (MCF 187 ± 29) relative to untreated cells (MCF 122 ± 27).

Another feature of the senescent cell phenotype is the accumulation of lipofuscin, which we analyzed with SenTraGor™, a highly lipophilic and biotinylated analog of Sudan Black B that is detectable with an anti-biotin antibody conjugated with AF488 [48,49,50]. TIGK cells were incubated with medium or 20 pg/mL Cdt for 3–7 days and processed as described in Section 2. As shown in Figure 2C, the MCF for control cells was 19.9 ± 4.0 (3 days), 21.5 ± 1.8 (4 days), and 28.5 ± 5.6 (7 days). In the presence of Cdt, fluorescence increased in a time-dependent manner: 28.0 ± 8.0 (3 days), 34.5 ± 2.3 (4 days), and almost doubling at day 7 to 47.8 ± 7.1. PGK cells were also assessed for lipofuscin following exposure to Cdt (Figure 2D); cells treated with 10 and 25 pg/mL Cdt exhibited increased fluorescence with MCFs of 41.2 ± 5.4 and 40.2 ± 4.0 compared to 25.8 ± 5.2 in control cells. Results from earlier time points are shown in Figure S2.

### 3.2. Cdt Induces Oral Epithelial Cells to Exhibit the Senescent Associated Secretory Phenotype (SASP)

**Figure 3:**
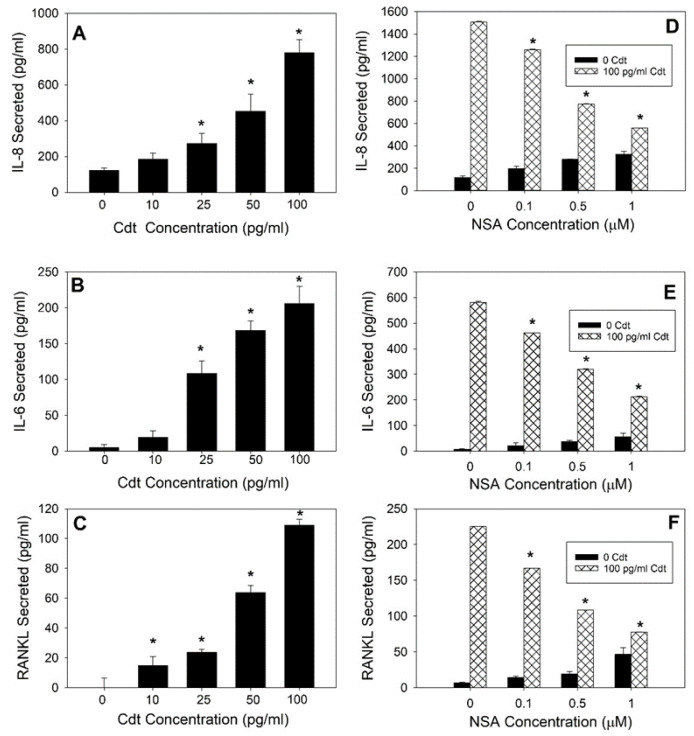
Cdt induces SASP in TIGK cells. TIGK cells were treated with Cdt (0–100 pg/mL) for 72 h; cell supernatants were harvested and analyzed by ELISA for release of IL-8 (panel (**A**)), IL-6 (panel (**B**)) and RANKL (panel (**C**)). In a second series of experiments, TIGK cells were pre-treated with the GSDMD inhibitor NSA (0–1 μM) for one h, followed by the addition of Cdt (100 pg/mL). Supernatants were harvested 72 h later and analyzed for release of IL-8 (panel (**D**)), IL-6 (panel (**E**)) and RANKL (panel (**F**)). Results are the mean ± SEM of three experiments; * indicates statistical significance (*p* < 0.01).

In addition to both enhanced SA-β-gal activity and lipofuscin, senescent cells typically acquire a phenotype characterized by robust secretory activity known as SASP [46,47,51]. This secretory phenotype has been identified in almost all senescent cells and may include pro-inflammatory cytokines, chemokines, proteases and other biologically active agents. It is in this context that we next assessed Cdt-treated TIGK cell supernatants for IL-8 (Figure 3A), IL-6 (Figure 3B) and RANKL secretion (Figure 3C). All three cytokines exhibited dose-dependent increases when cells were exposed to 0–100 pg/mL Cdt. IL-8 secretion increased from 123.1 ± 13.8 pg/mL in control cells to 184.8 ± 35.5, 273.1 ± 55.7, 453.3 ± 95.1 and 780 ± 73.0 pg/mL in the presence of 10, 25, 50 and 100 pg/mL Cdt, respectively. Similarly, IL-6 levels increased from 4.7 ± 4.5 pg/mL in control cells to 19.2 ± 8.9, 108.7 ± 17.2, 168.3 ± 13.6 and 206.2 ± 23.6 pg/mL in the presence of the same doses of toxin (10–100 pg/mL). Significant increases in RANKL secretion were also observed in a dose-dependent manner; in the presence of 10, 25, 50 and 100 pg/mL, Cdt RANKL levels were 15 ± 6, 23.9 ± 2.0, 63.9 ± 4.5 and 108. ± 4.0 pg/mL, respectively. No detectable RANKL was observed in the supernatants of control cells.

Recent studies have suggested that gasdermin D (GSDMD) activation is a key event leading to the release of senescence-associated proteins [52,53,54,55,56,57]. GSDMD is converted to an active fragment (GSDMD-NT), which causes nonlytic membrane pore formation. Small GSDMD-NT-dependent pores have recently been observed in a variety of senescent cells and are now considered a significant feature of cellular senescence [58]. To ascertain if GSDMD activation was involved in Cdt-induced cytokine release from TIGK cells, we employed the GSDMD inhibitor necrosulfonamide (NSA); results are shown in Figure 3D–F. Cells were pre-treated with 0.1–1.0 μM NSA for 60 min, followed by the addition of 100 pg/mL Cdt. Supernatants were analyzed by ELISA and demonstrated that IL-8, IL-6 and RANKL levels were reduced in a dose-dependent manner when cells were pretreated with 0.1, 0.5 and 1.0 μM NSA. Maximum inhibition occurred in the presence of the highest NSA dose employed in this study (1.0 μM), with all three cytokine levels reduced by >60%. Interestingly, we observed small increments of cytokine release in the presence of NSA alone; this was not due to NSA-induced changes in either cell viability or induction of senescence (Figure S3).

### 3.3. Cdt-Induced Senescent PGKs Exhibit Loss of Barrier Function

**Figure 4:**
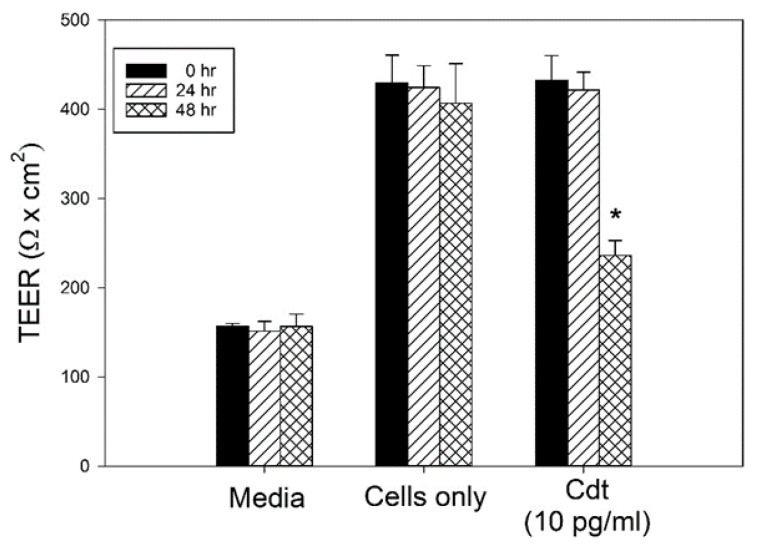
Cdt-induced OE senescent cells exhibit a breakdown in epithelial barrier function. PGKs were grown to confluence until a stable TEER was established. Medium or Cdt was then added and the cells assessed daily for changes in TEER as described in Section 2. Results of three experiments were plotted as mean resistance (Ω × cm^2^) at 24 h (solid bars), 48 h (hatched bars) and 72 h (cross hatched bars); * indicates statistical significance (*p* < 0.01).

We next assessed Cdt-treated cells for alterations in epithelial barrier function. For this purpose, we measured transepithelial electrical resistance (TEER), which is a widely accepted quantitative technique to measure the integrity of cellular barriers in epithelial cell culture models [44]. This technique categorizes cell barriers as tight, intermediate, or leaky based upon TEER values. We have demonstrated that PGK monolayers reproducibly develop TEER with intermediate values (429 ± 30.9 Ω × cm^2^; Figure 4); moreover, after 48 h exposure to Cdt, the TEER values were significantly reduced to essentially the background levels observed with medium alone (236 ± 16.6 Ω × cm^2^).

**Figure 5:**
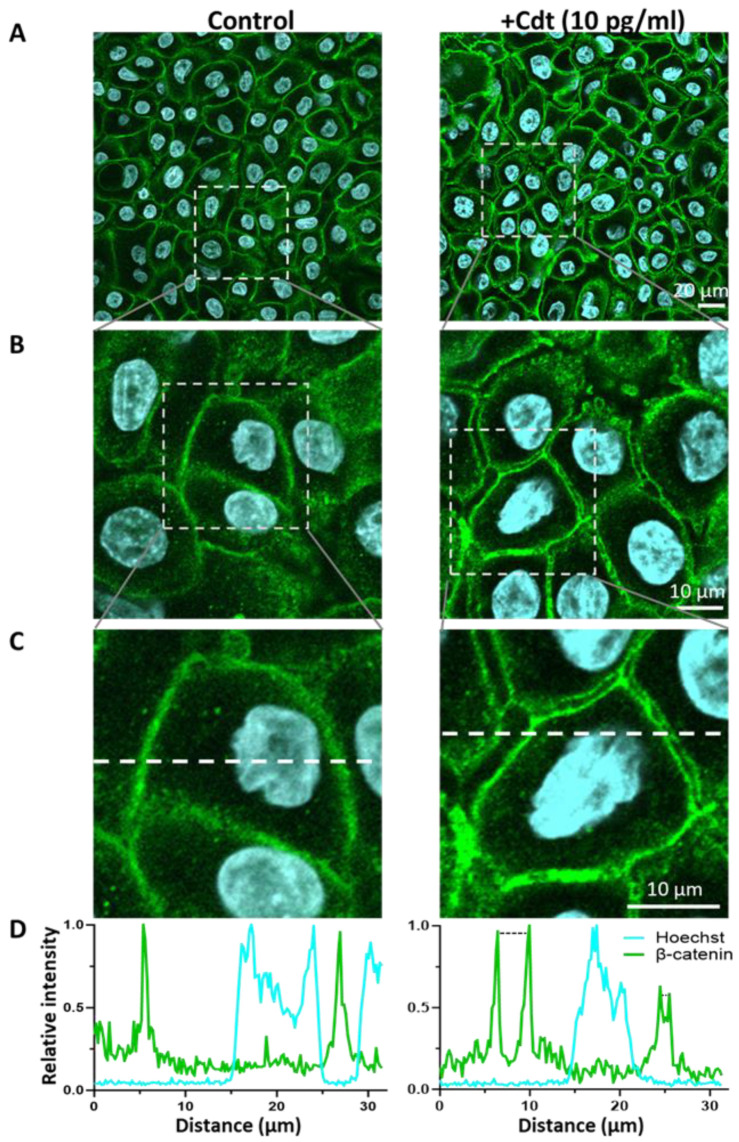
Cdt treatment alters cell–cell contacts in PGKs. (**A**) Confocal images showing control (untreated) and Cdt (10 pg/mL, 48 h)-treated PGKs immunostained with ß-catenin (green). Nuclei stained with Hoechst are pseudo-colored in cyan. (**B**) Boxed regions in the panel (**A**) were enlarged and shown. (**C**) The boxed regions in panel (**B**) were further enlarged, highlighting the appearance of distinct gaps between cells in the Cdt-treated set (right) relative to the control cells. (**D**) Line intensity profiles for ß-catenin (green) and Hoechst nuclear stain (cyan) across the white dotted line in panel (**C**). The gaps between the adjacent cells identified by the ß-catenin staining pattern are depicted by dotted black lines.

To further evaluate whether Cdt perturbs epithelial barrier function, cell–cell adhesion in PGKs was examined by immunostaining Cdt-treated (10 pg/mL) and untreated (control) cells for an adherans junction protein, ß-catenin [59]. There was distinct staining for ß-catenin adjoining the border between adjacent cells in the control cultures (Figure 5A–C left, Figure S4 top). Treatment of PGK cells with 10 pg/mL Cdt (48 h) resulted in an alteration of the staining pattern for ß-catenin; intense staining outlining the individual cells was detected while the junctions between the cells were largely devoid of ß-catenin (Figure 5A–C right, Figure S4 bottom). The linear intensity profile (Figure 5D) further highlights these ß-catenin negative gaps (1.45 ∀ 0.25 µm; size ranging from 0.4–2.9 µm based upon analysis of 10 regions) between cells treated with Cdt. This result strongly points to Cdt-driven disruption of adherans junctions that can lead to compromised cell–cell communication and compromised barrier function.

### 3.4. Cdt-Induced Cellular Senescence Is Dependent on the Host Cell Protein Cellugyrin

**Figure 6:**
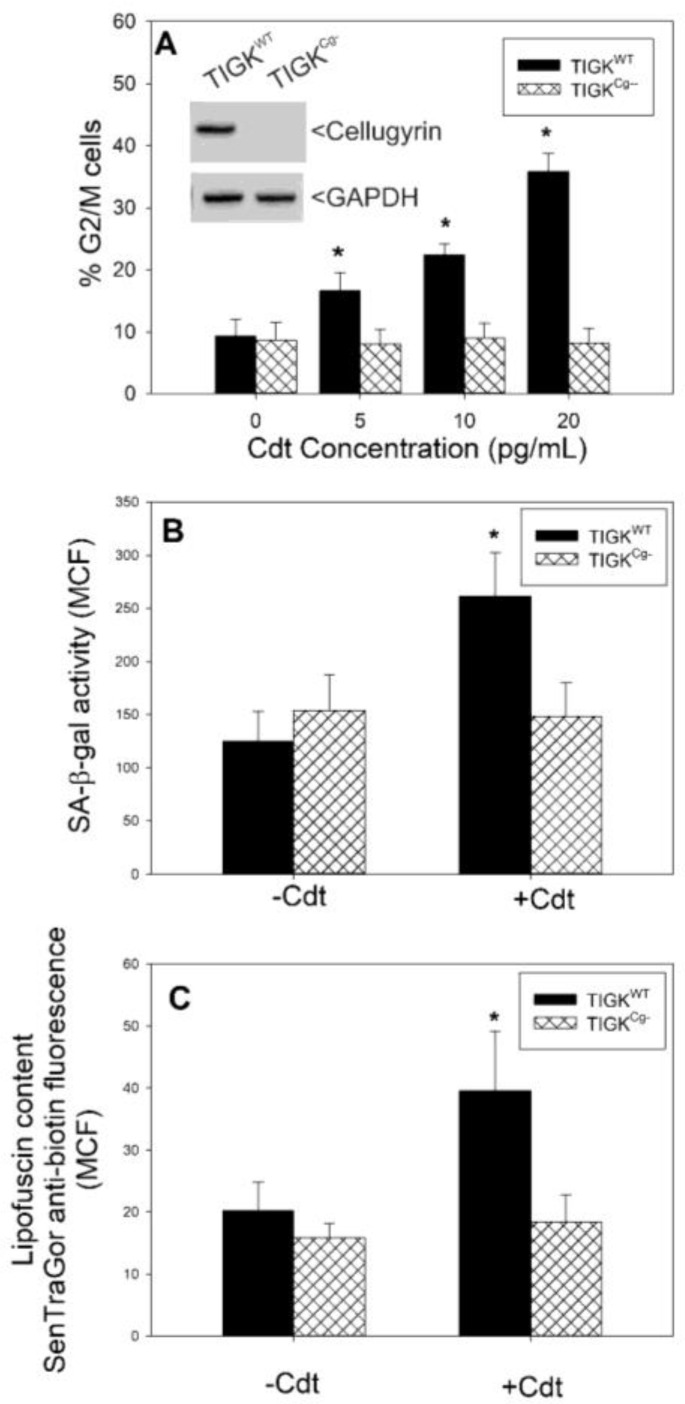
Cdt-induced cellular senescence is dependent on the host cell protein cellugyrin. Cellugyrin-deficient TIGK cells (TIGK^Cg−^) were prepared using CRISPR/Cas9 gene editing (inset panel (**A**)). In panel A, TIGK^Cg−^ (cross-hatched bars) were compared with TIGK^WT^ cells (solid bars) for susceptibility to Cdt-induced cell cycle arrest. The percentage of G2/M cells was determined using propidium iodide fluorescence and flow cytometry; the results are plotted as the percentage of G2/M cells (mean ± SEM) versus Cdt concentration. Panel (**B**) compares the effect of Cdt (10 pg/mL) on TIGK^WT^ and TIGK^Cg−^ cell SA-β-gal activity after 72 h; the data are plotted as SA-β-gal fluorescence [MCF; (mean ± SEM)]. Panel (**C**) shows the effect of Cdt on lipofuscin content in TIGK^WT^ and TIGK^Cg−^ cells following 96 h exposure to the toxin; results are plotted as lipofuscin content [MCF; (mean ± SEM)]. * indicates statistical significance (*p* < 0.05) when compared to similarly treated TIGK^WT^ cells.

In previous studies with lymphocytes and macrophages, we demonstrated that shortly after Cdt binds to cholesterol within lipid-enriched membrane microdomains, the host cell protein, cellugyrin, localizes to the same region. Following these events, Cdt subunits CdtB and CdtC are internalized and associate with cellugyrin, leading to their intracellular transport complexed to cellugyrin within the context of synaptic-like microvesicles (SLMVs) [35,36,38]. Importantly, reduced expression of cellugyrin in lymphocytes and macrophages protects cells from CdtB internalization and subsequent toxicity. Therefore, we assessed whether the susceptibility of TIGK cells to Cdt-induced senescence was also dependent on cellugyrin. CRISPR/Cas9 gene editing was employed using a pLentivirus V2 plasmid containing cellugyrin-specific sequences to establish a cellugyrin-deficient TIGK cell line (TIGK^Cg−^). As shown in Figure 6A inset, TIGK^Cg−^ cells do not express detectable cellugyrin. Wildtype TIGK cells (TIGK^WT^) and TIGK^Cg−^ cells were treated with Cdt for 48 h and assessed for cell cycle arrest (G2/M). Similar to our previous results [39], TIGK^WT^ cells exhibited a dose-dependent response to Cdt, as the accumulation of G2/M cells was 16.6 ± 3.0%, 22.4 ± 1.9% and 35.8 ± 2.9% following exposure to 5, 10 and 20 pg/mL Cdt, compared to 9.3 ± 2.8% in untreated controls. In contrast, TIGK^Cg−^ cells were protected from Cdt-induced cell cycle arrest as the percentage of cells in the G2/M phase did not change from those values observed in control cells (8.6 ± 2.9%).

We then evaluated the susceptibility of TIGK^Cg−^ cells to Cdt-induced increases in SA-β-gal activity and lipofuscin accumulation. TIGK^WT^ and TIGK^Cg−^ cells were treated with 20 pg/mL Cdt, followed by analysis 72 h later for SA-β-gal activity as described earlier. TIGK^WT^ cells exhibited an increase in SA-β-gal activity (Figure 6B); MCF increased from 125 ± 27.5 in control cells to 261.4 ± 41.1 in Cdt-treated cells. In contrast, Cdt-treated TIGK^Cg−^ cells did not exhibit any increase in fluorescence relative to control cells; the MCFs were 148.9 ± 31.8 (Cdt-treated) and 153.7 ± 34.1 (control). In a similar experiment, TIGK^WT^ and TIGK^Cg−^ cells were treated with Cdt and assessed 96 h later for changes in lipofuscin using the SenTraGor reagent and anti-biotin antibody conjugated to AF488 (Figure 6C). Toxin-treated TIGK^WT^ cells exhibited an increase in fluorescence, as MCFs were 20.3 ± 4.5 in control cells and 39.5 ± 9.7 in toxin-treated cells. Cdt-treated TIGK^Cg−^ cells failed to exhibit a change in fluorescence relative to control cells. These results confirm that, similar to other cell types examined to date, the susceptibility of OEs to Cdt is also dependent upon cellugyrin [35,36,38].

## 4. Discussion

Durable cell cycle arrest in the absence of cell death is a hallmark of cellular senescence; in this state, cells are no longer proliferating but remain metabolically active. Multiple types of cellular senescence have been described, including replicative, oncogenic, genotoxic and developmental, among others [60,61,62]. Moreover, the senescent phenotype has been implicated as a significant contributing factor to the pathogenesis of a wide range of disorders, including cancer, fibrosis, cardiovascular disease, diabetes, osteoarthritis and neurological disorders [46,63,64,65]. Recent studies have also demonstrated that pathogens, both bacterial and viral, are contributors to senescence [66,67]. Indeed, infection-induced senescence has been shown to involve toxins, viral capsids and flagella [67,68,69,70,71]. It should be noted that several studies have also demonstrated that bacterial factors such as *Porphyromonas gingivalis* lipopolysaccharide (LPS) can induce senescence in fibroblasts and dendritic cells, thereby contributing to periodontal disease-associated inflammation [72,73,74,75].

In this study, we demonstrated that, in addition to sustained G2/M arrest, *Aa*Cdt-treated OEs exhibit two of the most common hallmarks of senescent cells: elevated levels of SA-β-gal activity and the accumulation of oxidatively modified proteins and degraded lipid, commonly referred to as lipofuscin. Perhaps one of the most interesting, if not significant, features of senescent cells is that they also produce a secretome known as SASP, which contains pro-inflammatory mediators among other biologically active agents [46,47]. We have demonstrated that Cdt-treated OEs secrete IL-6, IL-8 and RANKL 72 h following exposure to toxin while remaining suspended in the G2/M phase of the cell cycle. Additionally, cytokine secretion exhibited dependence on GSDMD activation as the GSDMD inhibitor, NSA, blocked the toxin-induced release of all three cytokines. It should be noted that GSDMD activation as well as the formation of GSDMD-mediated nonlytic pores have recently been shown to be a feature shared by many senescent cells and are critical to senescence-associated secretory protein release [52,53,54].

The SASP is, perhaps, the most significant aspect of cellular senescence from a pathogenic perspective. The upregulated secretion of generally pro-inflammatory mediators allows senescent cells to: (1) induce senescence in neighboring cells (paracrine senescence), thereby further enhancing SASP secretome production and reducing the population of normal functioning (healthy) cells in involved tissue; (2) alter the local environment within the tissue, perhaps contributing to increased susceptibility to infection; and (3) promote recruitment and activation of inflammatory cells (reviewed in [46,47,67]. Thus, induction of cellular senescence represents a cell function vulnerable to hijacking and exploitation by pathogens; in this scenario, cellular senescence and SASP contribute to sustained infection and chronic inflammation [67]. Moreover, we demonstrate that senescent OEs exhibit another unique feature: breakdown of barrier function. Clearly, Cdt-induced cellular senescence can have a profound effect on oral epithelial tissue and contribute to disease initiation and/or progression. This axis of cellular toxicity may account for the significant role that *A. actinomycetemcomitans* has been reported to play in the conversion from periodontal health to disease, as has been observed for MIPP (previously reported as LAP) (reviewed in [1,2,6]). While direct evidence of a role for senescence in periodontal disease is currently limited, there is increasing acknowledgment of its potential participation in age-associated alterations within the periodontal environment as it relates to disease susceptibility [51,76,77]. Investigators have also demonstrated the presence of senescent osteocytes obtained from the alveoli of old mice, suggesting that these cells contribute to alveolar bone resorption [75]. As noted earlier, *P. gingivalis* LPS has also been reported to induce cellular senescence, further supporting the fact that the associated phenotypic changes are involved in the pathogenesis of periodontitis.

Senescence of various cells shares many attributes; however, it should be noted that they are not identical, as many of their novel features are dependent upon cell type and the pathway leading to senescence [45,62,78]. In this context, it is important to note that *Aa*Cdt-induced senescence in OEs is unique as it occurs in the absence of DNA damage [39]. Moreover, *Aa*Cdt-induced senescence is associated with a novel mechanism involving PI-3K signaling blockade and concomitant GSK3β activation, which mediates downstream phosphorylation of CDK1 (inactivation) [39]. *Haemophilus ducreyi* Cdt has been shown to also induce senescence in several cell lines [60,79,80,81]. In these studies, cells were exposed to relatively higher doses of toxin relative to those used in our current study, and senescence was associated with the activation of the DNA damage response.

Another unique feature of Cdt-induced toxicity, in general, and Cdt-induced senescence, in particular, is the dependence on the host cell protein cellugyrin (synaptogyrin-2). This protein belongs to the synaptogyrin family, a group of proteins that contain four transmembrane regions with a tyrosine-phosphorylated tail. Within cells, cellugyrin is embedded in SLMV^Cg+^ [35,36], which likely serve as sorting vesicles. These structures contain proteins essential for endocytic processing and are likely components of the trans-Golgi network (TGN) [82,83]. We have previously shown that both the CdtB and CdtC subunits are associated with cellugyrin and that intracellular transport of the Cdt subunits in macrophages and lymphocytes is dependent upon this association [35,36]. Moreover, we now demonstrate that, in addition to lymphocytes and macrophages, this dependence on cellugyrin extends to OEs. Specifically, epithelial cells rendered unable to express cellugyrin, TIGK^Cg−^ cells, were protected from Cdt-induced cell cycle arrest and senescence.

It is generally accepted that cellular senescence evolved as a protective mechanism to eliminate cells containing damaged DNA that escaped both repair and elimination by apoptosis [61,63]. However, it is now clear that this protective response represents another cell function vulnerable to exploitation by pathogens. As noted earlier, the colonization of supragingival biofilms by *A. actinomyetemcomitans* is not sufficient to cause periodontitis but represents a risk factor for the onset of gingival inflammation. Now considered an accessory pathogen, *A. actinomycetemcomitans* eventually translocates from the gingival margin through the gingival epithelium into the underlying connective tissue; this transition is associated with the conversion from periodontal health to disease [1,2,6]. There is extensive literature demonstrating a role for Cdt in the pathogenesis of disease, attributed to the wide range of Cdt-producing pathogens that are associated with sustained infection and promotion of inflammation in mucocutaneous tissues (reviewed in [11]). Moreover, evidence is accumulating to link *A. actinomyetemcomitans-*associated periodontitis with Cdt; for example, several studies demonstrate that a high percentage of *A. actinomyetemcomitans* isolates from LAP-diseased sites contain the *cdt* genotype and/or express active toxin [84,85,86,87]. There is growing evidence from studies employing human gingival explant models as well as in vivo animal models that demonstrate the ability of Cdt to induce cell cycle arrest, disrupt the epithelial barrier and penetrate the epithelium [88,89,90]. Ohara et al. [90] observed that Cdt-mediated cell cycle arrest occurs in vivo within basal cells of junctional and gingival epithelium; they propose that cell cycle blockade contributes to subsequent desquamation and detachment of junctional epithelial cells. Our current study is consistent with these observations, as it demonstrates that an important feature of the Cdt-induced epithelial senescent phenotype is associated with disruption of epithelial barrier function. Our TEER data and fluorescence microscopic analysis of epithelial cells indicate that this likely involves disruption of adherens junctions containing β-catenin.

**Figure 7:**
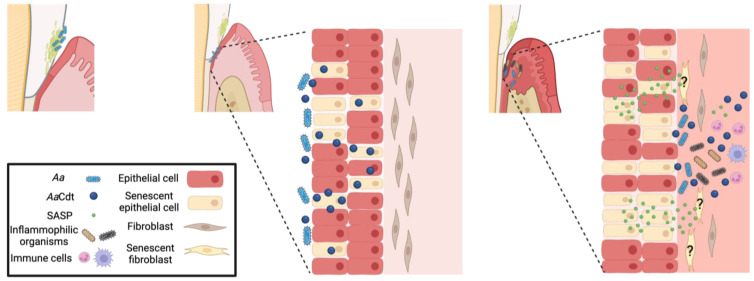
Model depicting the role of *Aa*Cdt-induced senescence in the pathogenesis of MIPP. The left panel shows healthy tissue at risk for MIPP due to the presence of supragingival *A. actinomycetemcomitans* (*Aa*). Initial exposure to *Aa*Cdt occurs while the bacteria are at the gingival margin, leading to cell cycle arrest and senescence within the epithelium and concomitant loss of barrier function indicated as distinct gaps between epithelial cells (middle panel). Continued exposure to Cdt along with OE-derived SASP-associated proinflammatory mediators further contribute to increased OE senescence and translocation of *A. actinomycetemcomitans* into the subgingival tissue (right panel); collectively, the mediators contribute to an altered gingival microenvironment conducive to supporting infection by inflammophilic organisms. Noteworthy, continued exposure to *Aa*Cdt and/or SASP perpetuates the induction of OE senescence (and possibly fibroblasts) in the face of constant epithelial turnover. Ultimately, these events lead to the recruitment of both innate and acquired immune cells, chronic inflammation and bone destruction.

To date, we have demonstrated that Cdt is a tri-perditious toxin that can profoundly affect acquired and innate immune cells as well as epithelial barrier function [27,28,30,39,43,91,92]. We propose that *Aa*Cdt-induced OE senescence is a significant contributing factor to *A. actinomycetemcomitans* infection and the subsequent development of chronic inflammation. As outlined in Figure 7, the initial effect of epithelial exposure to Cdt likely occurs while *A. actinomycetemcomitans* is present at the gingival margin. We propose that local secretion (or release within outer membrane vesicles) of the toxin leads to cell cycle arrest within the epithelium. Further acquisition of the senescent phenotype leads to breakdown of the epithelial barrier, which we propose enables *A. actinomycetemcomitans* to transit the epithelium and colonize the underlying connective tissue. Additionally, we suggest that sustained release of both the Cdt and SASP secretomes in this new location modifies the tissue such that it becomes supportive of not only *A. actinomycetemcomitans* infection but also of other inflammophilic organisms. Over time, the collective effects of sustained SASP, microbial infection and Cdt release contribute to a pro-inflammatory state. It is noteworthy that Belibasakis et al [93,94] previously reported that Cdt induces cell cycle arrest and IL-6 release from gingival fibroblasts; they did not relate these observations to senescence. As noted above, one of the unique properties of SASP is its ability to promote senescence in otherwise healthy adjacent cells. This property allows for both the amplification of senescence and the continued induction of senescence in the face of continual epithelial cell turnover. These events ensure that senescent cells remain continually present in the diseased tissue and that the altered environment contributes to the chronicity of *A. actinomycetemcomitans* infection and inflammatory disease.

In conclusion, the involvement of cellular senescence, in general, and pathogen-induced cellular senescence, in particular, in periodontal disease pathogenesis has several clinical implications. For example, these Cdt-mediated pathologic events account for many of the virulence characteristics ascribed to *A. actinomycetemcomitans*, including tissue invasiveness, the ability to create an environment that facilitates the accumulation of other organisms, the evasion of host defenses and the ability to promote inflammation. Moreover, senescent cell involvement in disease pathogenesis offers new opportunities for disease classification, diagnosis and therapy.

## Data Availability

Data are provided within the context of this article.

## Supplementary Materials

The following supporting information can be downloaded at: https://www.mdpi.com/article/10.3390/pathogens13020155/s1, Figure S1: Representative cell cycle histogram profiles; Figure S2: Lipofuscin content in TIGK cells at 24 and 48 h; Figure S3: Viability and SA-β-gal activity assessed in TIGK cells exposed to Cdt and NSA; Figure S4: Tiled view of the confocal z-scan.
