# Supplementary material for: Aggregatibacter actinomycetemcomitans Cytolethal Distending Toxin Induces Cellugyrin-(Synaptogyrin 2) Dependent Cellular Senescence in Oral Keratinocytes
Source: Pathogens. 2024 Feb 8;13(2):155. doi: 10.3390/pathogens13020155 (PMC10892517; doi:10.3390/pathogens13020155)
Supplement: Supplementary file 1 [file pathogens-13-00155-s001.zip › pathogens-2810356-supplementary.pdf]

Supplemental Figures

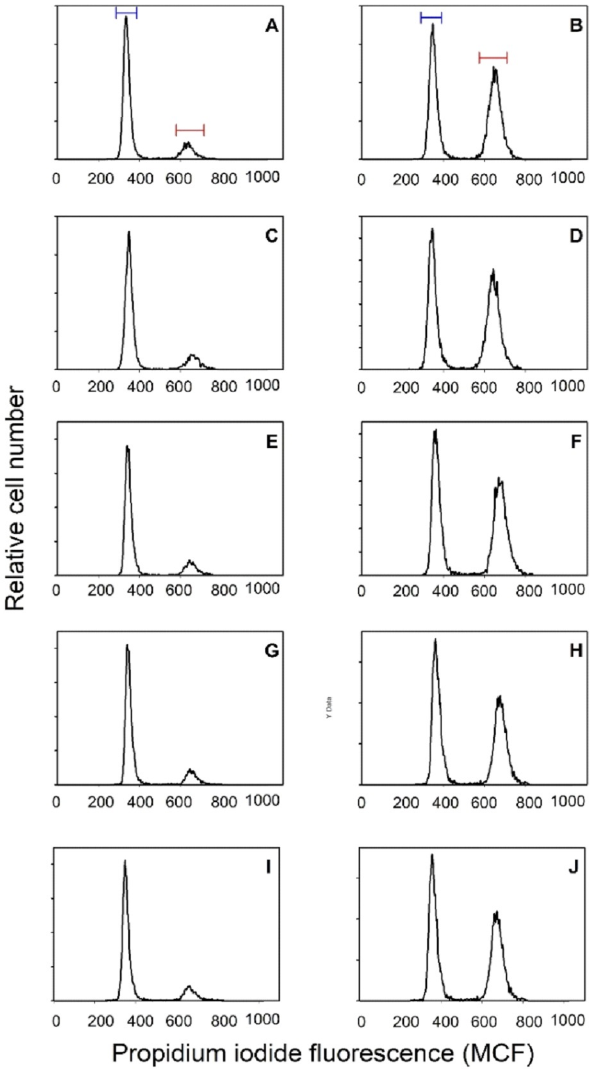

Figure S1: Representative histogram profiles are shown for the cell cycle data presented in Fig 1D using propidium iodide fluorescence to determine cell cycle distribution. Panels A, C, E, G, I show representative results of control cells (medium alone) incubated for 3-7 days, respectively. Panels B,D,F,H and J show histogram profiles for cells incubated with Cdt for 3-7 days respectively. Analytical gates used to detect cells in the G0/G1 phases (blue) and G2/M phase (red) are shown in panels A and B. Cells with propidium iodide fluorescence between these two gates were designated as S-phase

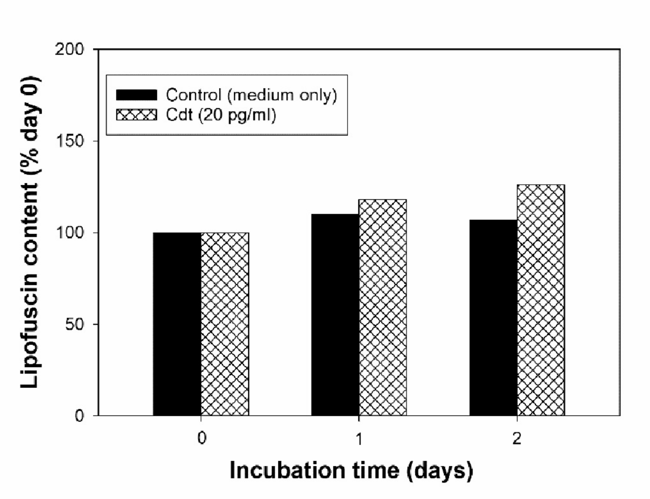

Figure S2. Lipofuscin content in TIGK cells at 24 and 48 hrs. TIGK cells were incubated with medium alone or with Cdt (20 pg/ml) for 0, 24 or 48 hr and the lipofuscin content determined as described in Section S2. Data are presented as lipofuscin (MCF) as percentage of day 0 values.

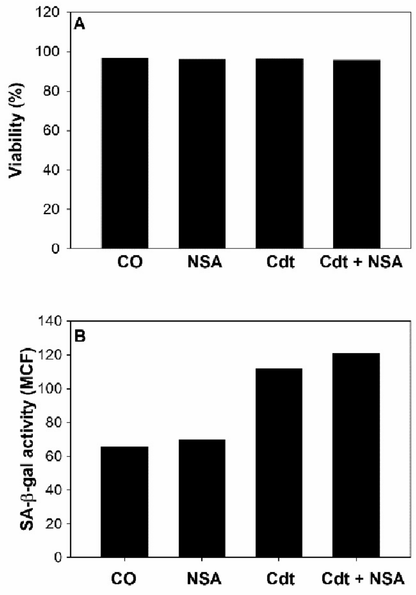

Figure S3. Viability and SA-β-gal activity were assessed in TIGK cells exposed to Cdt and NSA. TIGK cells were incubated with medium alone (CO), NSA (1 μM), Cdt (20 pg/ml) or both NSA and Cdt for 72 hrs. Panel A shows the viability of cells at 72 hrs determined by propidium iodide uptake and analyzed by flow cytometry; results are expressed as % viability. Panel B shows the levels of SA-β-gal activity at 72 hr expressed as MCF.

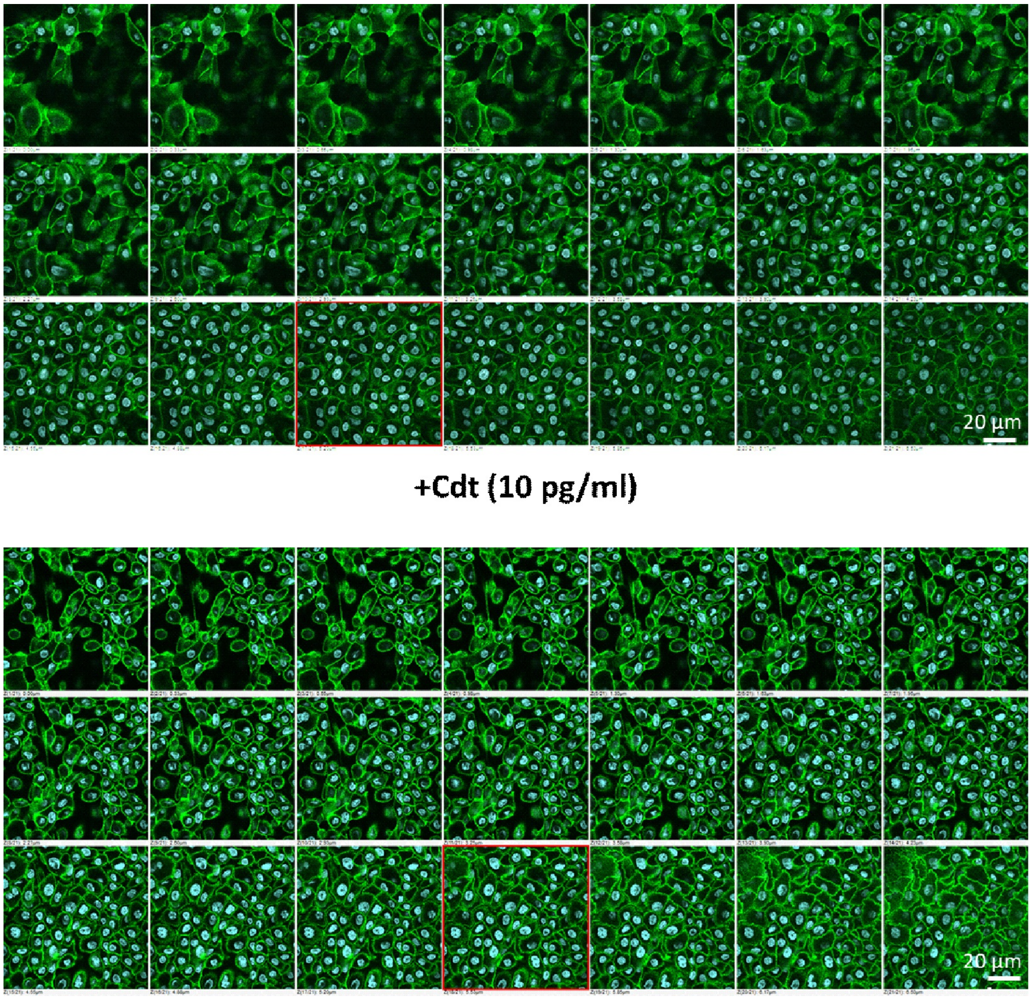

Figure S4: Tiled view of the confocal z-scan (step size 0.33 µm) from control (top) and Cdt (10 pg/ml; bottom) treated PGK immuno-stained for ß-catenin (green). Nuclei are pseudo-colored in cyan. Slices shown in Figure 5 have been outlined in red.
